# A multicenter cross-sectional survey of the role of community pharmacists, attitudes, and perceptions in preventing and controlling cardiovascular diseases

**DOI:** 10.1371/journal.pone.0314487

**Published:** 2025-02-10

**Authors:** Nthabiseng Florina Motlohi, Kofi Boamah Mensah, Neelaveni Padayachee, Ruwayda Petrus, Saleh F. Alqifari, Varsha Bangalee

**Affiliations:** 1 Discipline of Pharmaceutical Sciences, University of KwaZulu-Natal, Durban, South Africa; 2 Faculty of Pharmacy and Pharmaceutical Sciences, Kwame Nkrumah University of Science and Technology, Kumasi, Ghana; 3 Department of Pharmacy and Pharmacology, University of Witwatersrand, Johannesburg, South Africa; 4 Discipline of Psychology, University of KwaZulu-Natal, Durban, South Africa; 5 Department of Pharmacy Practice, University of Tabuk, Tabuk, Saudi Arabia; King Faisal University, SAUDI ARABIA

## Abstract

**Background:**

Cardiovascular diseases are a leading cause of mortality globally and impose suffering and economic difficulties, particularly in low- and middle-income countries. Community pharmacists present an opportunity for effective prevention and control of cardiovascular diseases. The overarching aim of the study was to evaluate factors associated with the extent of involvement, barriers and facilitators, and perceptions of Lesotho community pharmacists in preventing and controlling cardiovascular diseases.

**Methods:**

The study utilised a quantitative cross-sectional survey. A semi-structured questionnaire was distributed to licensed community pharmacists across four districts between March and July 2023. Parametric and non-parametric tests were performed for data analysis using a Statistical Package for Social Sciences version 26.

**Results:**

Apart from medicine dispensing, community pharmacists were mostly involved in hypertension (mean = 4.38±.73) and diabetes (mean = 4.17±.91) screening, weight management advice (mean = 3.81±.87), disease education (mean = 3.93±.83), medication management therapy (mean = 3.74±.99, 3.81±.88), referral of and follow up on patients (mean = 3.70±.98 and 3.87±.92). There was a significant association between the extent of involvement and pharmacy location, experience of community pharmacists, availability of tools, number of patients seen daily, and presence of other healthcare professionals at a community pharmacy (p<0.05). The most common barriers were related to patient factors (>75% agree to strongly agree), such as lack of awareness of community pharmacists’ services. Community pharmacists possessed positive (mean >3) attitudes and perceptions regarding their role in cardiovascular disease management.

**Conclusions:**

Besides dispensing medicine, community pharmacists had varying extent of involvement in health promotion activities. The provision of these services differed between socio-demographic groups. Community pharmacists possessed good knowledge, positive attitudes towards their cardiovascular disease management role. Thus, they can improve cardiovascular disease outcomes. However, the barriers potentially limit their scope of practice and encourage inconsistent community pharmacy services. The findings present pertinent information to policy-makers, regulators, and pharmacists that can inform the development of frameworks to improve clinical and pharmacy practice in Lesotho and low- and middle-income countries.

## Background

Cardiovascular diseases (CVDs) are a group of non-communicable diseases of the heart and blood vessels [1]. CVDs are a leading cause of mortality globally. Approximately 18 million deaths occur annually due to CVDs globally [2, 3]. Strikingly, 33% of affected populations are younger than 70 years, thus imposing suffering and economic difficulties, particularly in low- and middle-income countries (LMICs), which carry over 75% (n = 13 million) of CVDs-related global mortality [2, 3]. Over the past two decades, a substantial decline in mortality was realised in high-income countries (HICs) through improved prevention and treatment strategies. In contrast, LMICs suffer the highest CVDs burden, with rates twice as high as those of HICs in recent years [3]. The World Health Organization has warned of a projected global mortality increase of 27% by 2030, suggesting immense strain on health systems in the future.

Lesotho is a Sub-Saharan African country classified under LMICs [4]. The country faces a growing burden of CVDs, which are the leading cause of mortality among the non-communicable diseases. Between 1990 and 2015, Lesotho experienced over 60% increase in CVD burden [5]. In 2016, the country reported 32% of total mortality due to non-communicable diseases, with CVDs contributing 44% of total non-communicable disease-related deaths [6]. Moreover, in 2019, 45% of overall non-communicable disease-related mortality was reported, which represented a 13% increase in three years (2016–2019) [7]. The increase in disease occurrences significantly strains healthcare systems and subsequent poor health outcomes [8]. It is thus imperative that effective strategies for preventing and controlling CVDs should be a priority in Lesotho.

Management of CVDs includes the detection, screening, treatment, and provision of palliative care for the people who need it [9]. Effective strategies to reduce the burden of CVDs can be classified into population-wide interventions to reduce overall risk factor exposure, individual approaches to modify risk factors for high-risk individuals, and treatment of CVD events, with the last two being applicable for the primary healthcare (PHC) setting [3]. At the PHC, three additional stages have been proposed to manage CVDs: primary prevention, secondary prevention, and early detection [3]. Cardiovascular diseases are highly preventable. The impact of CVDs can be minimised by addressing identifiable and modifiable risk factors such as the use of tobacco, unhealthy diets and obesity, physical inactivity, and the harmful use of alcohol. The outcomes of these risk factors may present in patients as noticeable signs (e.g., high blood glucose, high blood lipids, high blood pressure, overweight, and obesity) that can be measured and identified early at PHC facilities. Various healthcare providers are crucial in mitigating the burden of CVDs in PHC. Community pharmacists are easily accessible health professionals and are well-positioned to contribute to the PHC of CVDs [10]. Thus, they present an opportunity for effective prevention and control of CVDs. Previous studies have shown effective community pharmacists’ interventions [11–13]. However, majority of the reported work is based in HICs, leaving a gap in the relevance and applicability of the findings in LMICs [14].

The government of Lesotho recognises community pharmacy as an essential part of the private-for-profit healthcare system that provides care and medicines [15]. A qualitative survey of Lesotho community pharmacists discovered that the role of community pharmacists in CVD care includes detecting CVD risk factors and health promotion services such as lifestyle counselling, disease education, and medication adherence counselling [14]. The current study builds on the previous findings of a convenient to a more generalisable sample of community pharmacists in urban, suburban, and rural areas of Maseru (the capital city) and other districts. It seeks to assess the association between the extent of provision of services by community pharmacists to CVD patients and independent variables such as the socio-demographic characteristics of participants and the pharmacy settings. Understanding Lesotho community pharmacists’ involvement in preventing and controlling CVDs is important to policy-makers, regulators, and clinical practice to enable informed decision-making and development of effective strategies for preventing and controlling CVDs. The current study is part of a large mixed methods study that investigates the role of community pharmacists in preventing and controlling CVDs. The results will guide the development of a conceptual framework for the integration of community pharmacists in the PHC model. The overarching aim of the study was to determine factors associated with community pharmacists’ provision of services in preventing and controlling CVDs in Lesotho. Specifically, the study sought to answer the following research questions:

1. What are the socio-demographic factors that are associated with community pharmacists’ involvement in preventing and controlling CVDs in Lesotho?
2. What are the major barriers and facilitators perceived by Lesotho community pharmacists in the prevention and control of CVDs in Lesotho?
3. What is the relationship between attitude and perceptions and the community pharmacists’ involvement in the prevention and control of CVDs in Lesotho?

## Methods

### Study design

The study utilised a quantitative cross-sectional survey to satisfy the study objectives. Quantitative research is based on positivist philosophical underpinnings. Positivism asserts that there is a single reality applicable in different settings and that reality can be influenced by other factors (independent variables) that can be modified to control reality [16]. The authors identified the roles of Lesotho community pharmacists in preventing and controlling CVDs utilising a qualitative interview of a convenient sample size [17]. The researchers found a quantitative study approach a suitable method to determine the level of involvement of and factors influencing community pharmacists’ provision of services to CVD patients.

### Study setting

Lesotho is a small land-locked country in Sub-Saharan Africa with a population of approximately 2 million people [18]. Geographically, Lesotho comprises mostly plateaus, hills, mountains (highlands), and lowlands where the capital city (Maseru) is located. Administratively, the country is divided into 10 districts. The majority of community pharmacies (90%) are established in four districts in the lowlands, namely, Maseru, Berea, Mafeteng, and Leribe [8]. The population of people living in these four districts is estimated at 1.3 million people (65% of the national population) [19]. The study included community pharmacies based in the Maseru, Berea, Mafeteng, and Leribe districts.

### Participant recruitment and sampling

A database of community pharmacies and responsible registered pharmacists was obtained from the Ministry of Health of Lesotho. The study population was pharmacists working in community pharmacies in Maseru, Mafeteng, Leribe, and Berea districts who were registered with Lesotho Medical, Dental, and Pharmacy Council and had at least 6 months of community pharmacy experience. Records showed that there were 71 community pharmacies registered with the Ministry of Health of Lesotho across the four districts in 2022. The minimum sample size was calculated using a standard formula for a known population size for a cross-sectional study (Ellen, 2012):

$$n\;=N÷(1+N{\left(e\right)}^{2}$$

where:

n = sample size of the target study population

N = total number of target population

e = error margin (at 0.05)

n = 71/(1+71(0.05)^2^)

= 60 participants.

Previous community pharmacy surveys reported a response rate of 44% in Iran, 83% in Lesotho, and 94% in Ethiopia [20–22]. Therefore, the authors included all 71 community pharmacies to accommodate non-response. This approach is supported by literature that advises a census sample size when the population size is less than 200 [23]. All 71 community pharmacists were considered to participate in the survey because of the small population size. A convenient sample of community pharmacists willing and available to participate in the study during a 5-month data collection period (March to July 2023) was realised [24]. For invitations to participate in the study, emails were sent to all 71 community pharmacists, followed by a phone call. Upon acceptance of the invitation, participants were requested to sign an informed consent form and to facilitate the signing of a gatekeeper’s agreement by the pharmacy owners.

### Inclusion and exclusion criteria

The study participants were community pharmacists who had at least 6 months of community pharmacy experience, working on a full-time or part-time contractual terms at a community pharmacy recognised by Lesotho’s Ministry of Health, and registered with Lesotho Medical, Dental, and Pharmacy Council (according to the Ministry of Health community pharmacy database). Community pharmacists who were pharmacy owners but were not directly working in the front shop or with patients, pharmacy technicians, non-pharmacy staff, and pharmacy students practising in community pharmacies were excluded from participating in the study, as well as community pharmacists who were not willing to participate.

### Data collection

A self-administered, semi-structured questionnaire was developed guided by Theory of Planned Behaviour (TPB) constructs [25]. The researchers utilized findings of a previous qualitative survey of Lesotho community pharmacists’ role in preventing and controlling CVDs [17]. A standard Heart Disease Fact Questionnaire was utilised for questions assessing knowledge of CVD risk factors by community pharmacists [26]. The questionnaire was composed of 8 sections and 25 questions covering questions about socio-demographic details and pharmacy settings, involvement of community pharmacists in preventing and controlling CVDs, knowledge of CVD risk factors, barriers and facilitators of health promotion services to CVD patients, community pharmacists’ attitudes and perceptions of their role in preventing and controlling CVDs, cost of health promotion services, and availability of tools. The questions were composed of closed-ended questions, including multiple choice questions, a 5-point Likert scale, true or false, and a binary (available or not available) scale for availability of tools. Other questions, such as the cost of services, were left open-ended. Open-ended responses were coded and grouped into categories. The reliability of the Likert scale was measured using the Cronbach’s alpha coefficient [27]. A Cronbach’s alpha coefficient of ≥0.7 was considered satisfactory [28].The questionnaires were distributed from 01^st^ March to 30^th^ July 2023 through email or hand-delivered at the community pharmacies based on the participants’ preferences. To enhance validity, the questionnaire was reviewed and approved by the study team of supervisors (VB) and co-supervisors (KBM, NP, and RP), all of whom have extensive experience in pharmacy, psychology, academics, and research. Both online and paper-based versions of the questionnaire were pilot-tested with a convenience sample of five pharmacists with backgrounds in academic and community pharmacy. Minor modifications were made to enhance the clarity of the questions. The principal investigator (NFM) holds a Bachelor of Pharmacy (honours) and a Master of Technology in Pharmaceutical Sciences degree and is a current Doctor of Philosophy student.

### Data analysis

Data were analysed using the International Business Machines Corporation (IBM) Statistical Package for Social Sciences (SPSS) version 26 [29]. Descriptive analysis was performed, and data were presented as distribution frequencies and percentages, mean and standard deviations for each variable, and median for cost of services. An independent sample *t*-test was performed to calculate the mean differences between the independent variable groups, and *p*<0.05 was set as a cut-off point for significant differences. The Likert scale results were presented as frequency and frequency percentages for each scale division and the mean and standard deviation. The mean scores for the extent of community pharmacists’ involvement in providing services to CVD patients were further classified as less involved (less than the weighted mean average) and highly involved (equal to or above the weighted mean average). Similarly, the mean scores for the barriers and facilitators were categorised as most common (equal to or above the weighted mean average) (and less common (less than the weighted mean average) relative to the weighted mean average. Community pharmacists ’ knowledge of CVD risk factors was calculated as the proportion of correct responses. "I do not know" responses were regarded as incorrect. Scores <50%, 50%-59%, and ≥70% demonstrated low, moderate, and good knowledge, respectively [30]. Attitudes and perceptions mean scores ≥3 were considered positive attitudes or perceptions, and negative attitudes or perceptions were regarded as mean scores <3 [31].

### Data management and storage

NFM captured data into a Microsoft Excel sheet [32] and performed three repeated checks to verify the correctness of captured details against paper-based questionnaires. Data from paper-based questionnaires were merged with data from online questionnaires in a Microsoft Excel sheet. The data were then imported to SPSS. Data were manipulated for consistency of entries. For instance, the currency unit was deleted from the cost of services and the value was left as a computer-readable digit/continuous data. Subjects were removed from the final data analysis if the proportion of missing data was ≥ 10% of the questions [33] or there was no data on the main outcome variable (type of service) or invalid responses (e.g., number of working hours in a week written as (8 am–6 am). While missing data may significantly affect results and their application to a larger population, the literature suggests an acceptable limit ranging from 5% to 10% [Schafer JL., 1999, as cited in 33, 34], while other authors argued that mechanisms and patterns of missing values guide the informed decision on handling the data [35]. Respondents with missing data from other variables were kept because missing data were found to be random (in terms of geographical locations) and less than 10% per variable based on the questionnaire completion criteria. The respondents were de-identified by allocating a code (e.g., CPM001) and removing information such as the name of the pharmacy and email addresses from the paper-based questionnaires. Data were stored in a password-protected computer and lockable metal cabinets for paper-based data.

### Ethical considerations

The study’s protocol was approved by the Humanities and Social Sciences Research Ethics Committee of the University of Kwazulu-Natal (Ref: HSSREC/00003205/2021) and the Research and Ethics Committee of the Ministry of Health of Lesotho (Ref: ID101-2021-Renew 01). A study information leaflet was emailed to participants to familiarise themselves with the study contents and their rights as participants. Additionally, the purpose of the study and an outline of the participants’ rights were provided on the introductory page of the questionnaire. All potential participants gave consent before participating in the study by signing a consent form or selecting "yes" to whether they agreed to participate in the study for paper-based and online questionnaires, respectively. Pharmacy owners granted permission by signing a gatekeeper’s agreement. Participation in the study was voluntary and depended on the willingness and availability of the participants.

## Results

### Sociodemographic characteristics of the participants and pharmacy setting

Only 62 of the 71 targeted community pharmacists were reachable through emails and/or phone calls. Nine emails were undelivered or not responded to, while some phone numbers were unreachable. The unreachable participants were found to be random (in terms of geographical area since other demographic variables were not known to the authors). The unavailable participants were from Maseru (6), Leribe (2) and Mafeteng (1) districts. Therefore, 62 questionnaires were distributed through emails or hand-delivered. Out of the 62 questionnaires, 50 were returned, resulting in an overall response rate of 80.6% (22% online, 78% paper-based). Two paper-based questionnaires were incomplete. These were a male (18–29 years) and a female (30–44 years) community pharmacists operating in Maseru and Leribe respectively. Therefore, 48 respondents were included in the final data analysis.

**Fig 1:**
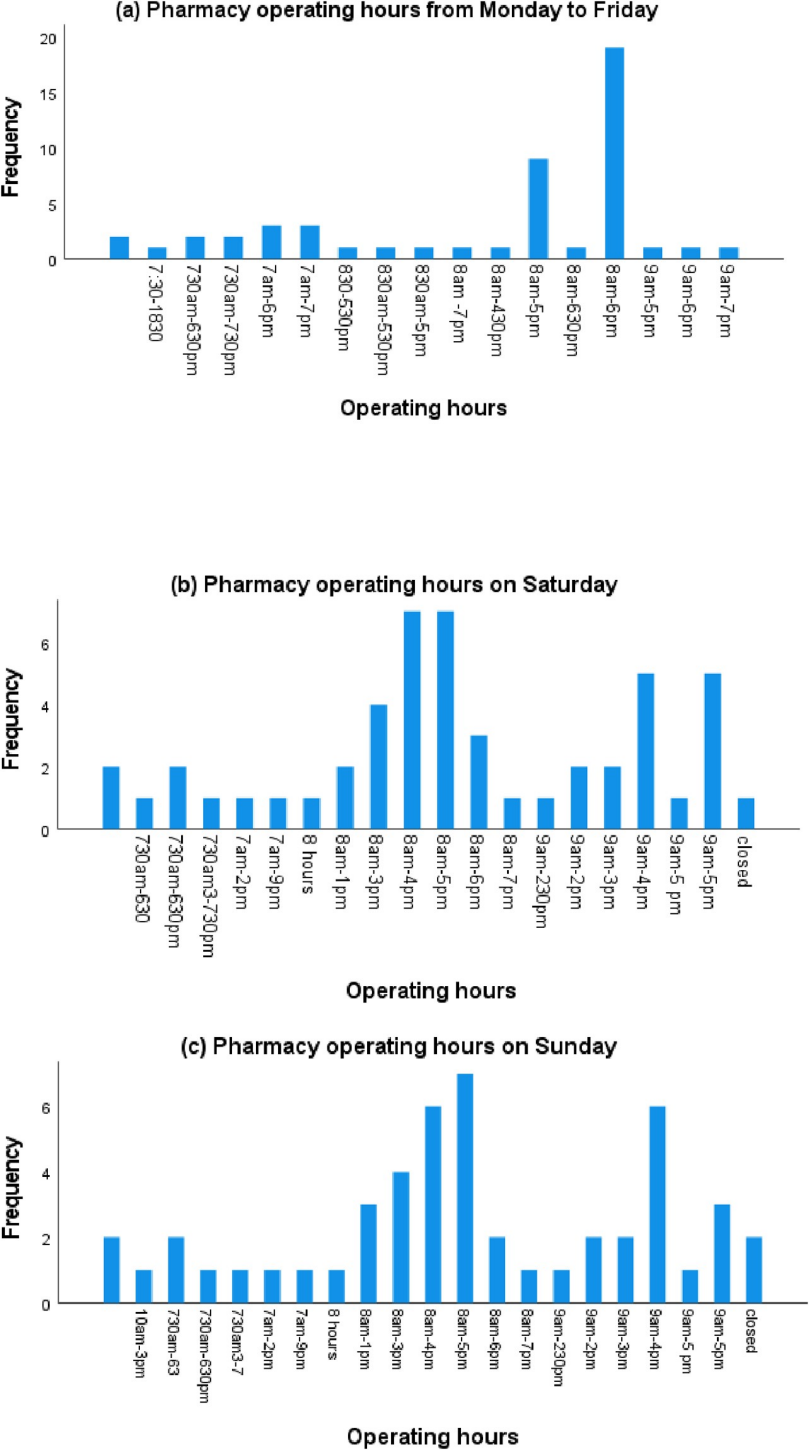
Community pharmacy weekly operating hours.

The socio-demographic details of the respondents and pharmacy operating hours are presented in Table 1 and Fig 1, respectively. More than half of the respondents were males (58.3%, n = 28), dominated by young pharmacists aged 18–29 years (60.4%, n = 29). Many of the respondents had 1 to 4 years of community pharmacy experience, while 10+ years was the least common number of years of experience (12.5%, n = 6). One pharmacist held a postgraduate qualification above the Bachelor of Pharmacy degree. All respondents received a CVD education during their pharmacy training, but the majority (83.3%, n = 38) had never received a related training postgraduate. Full-time employment was the most dominant status (81.2%, n = 39), either as employees (47.9%, n = 23) or pharmacy owners (33.3%, n = 16). Most respondents work for over 45 hours weekly. There was a more comparable representation of pharmacies in urban (43.7%, n = 21) and suburban (50.0%, n = 24) areas compared with rural area (6.3%, n = 3). Most pharmacies were independent stores with daily operating hours ranging from 8 to 12 hours during the week (Fig 1(a)). While 8 am to 6 pm was the most common operating hour, some community pharmacists open as early as 7 am and close late at 7 pm (Fig 1(a)). Most pharmacies operate with one pharmacist (72.9%, n = 35)) and at least one other healthcare professional (54%, n = 26)), with no general assistants (66.7%, n = 32).

**Table 1:** Socio-demographic characteristics of respondents (n = 48).

Variable	Category	Frequency	Valid frequency percentage (%)	Dichotomous groups
Gender	Female	20	41.7	Female
	Male	28	58.3	Male
Age group (years)	18–29	29	60.4	18–29
	≥30 and <60	19	39.6	≥30
Experience as a community pharmacist (years)	6 months to 1 year	10	20.8	<5 years
	1 year to 4 years	22	45.8	
	5 years to 9 years	10	20.8	≥5 years
	10+ years	6	12.5	
Level of education	Bachelor of Pharmacy/honours	47	97.9	Bachelor of Pharmacy/honours
	Masters in Pharmacy	1	2.1	Masters in Pharmacy
Received CVD education at undergraduate	Yes	48	100	Yes
	No	0	0	No
Continuous professional development in CVD management	Yes	8	16.7	Yes
	No	40	83.3	No
Employment status	Pharmacy owner and full-time employee	16	33.3	Pharmacy owner
	Pharmacy owner and part-time employee	2	4.2	
	Full-time employee	23	47.9	Employee
	Part-time employee	6	12.5	
Working hours per week (n = 45)	≤45 hours	11	24.4	≤45 hours
	>45 hours	34	75.6	≥45 hours
Location of the pharmacy	CBD/urban	21	43.7	CBD/urban
	Suburban (Within local public transport distance from the city)	24	50	
	Rural (outside of local public transport distance from the city)	3	6.3	Suburban/rural
Type of pharmacy	Independent	40	83.3	Independent
	Chain store	8	16.7	Chain store
District	Maseru (capital city)	35	72.9	Maseru
	Mafeteng	1	2.1	
	Berea	4	8.3	Other districts
	Leribe	8	16.7	
# of pharmacists	1 pharmacist	35	72.9	1 pharmacist
	>1 pharmacists	13	27.1	>1 pharmacist
Other healthcare professionals	No other healthcare professionals	22	45.8	No other healthcare professionals
	At least 1 other healthcare professionals	26	54.2	At least 1 other healthcare professionals
Availability of general assistants	No general assistants	32	66.7	No general assistants
	At least 1 general assistants	16	33.3	At least 1 general assistants
# of CVD patients seen/day	1–10 patients	28	58.3	1–10 patients
	≥11 patients	20	41.7	≥11 patients

CVD: Cardiovascular disease, CBD: Central business district.

### The extent of involvement of community pharmacists in preventing and controlling CVDs in Lesotho

**Fig 2:**
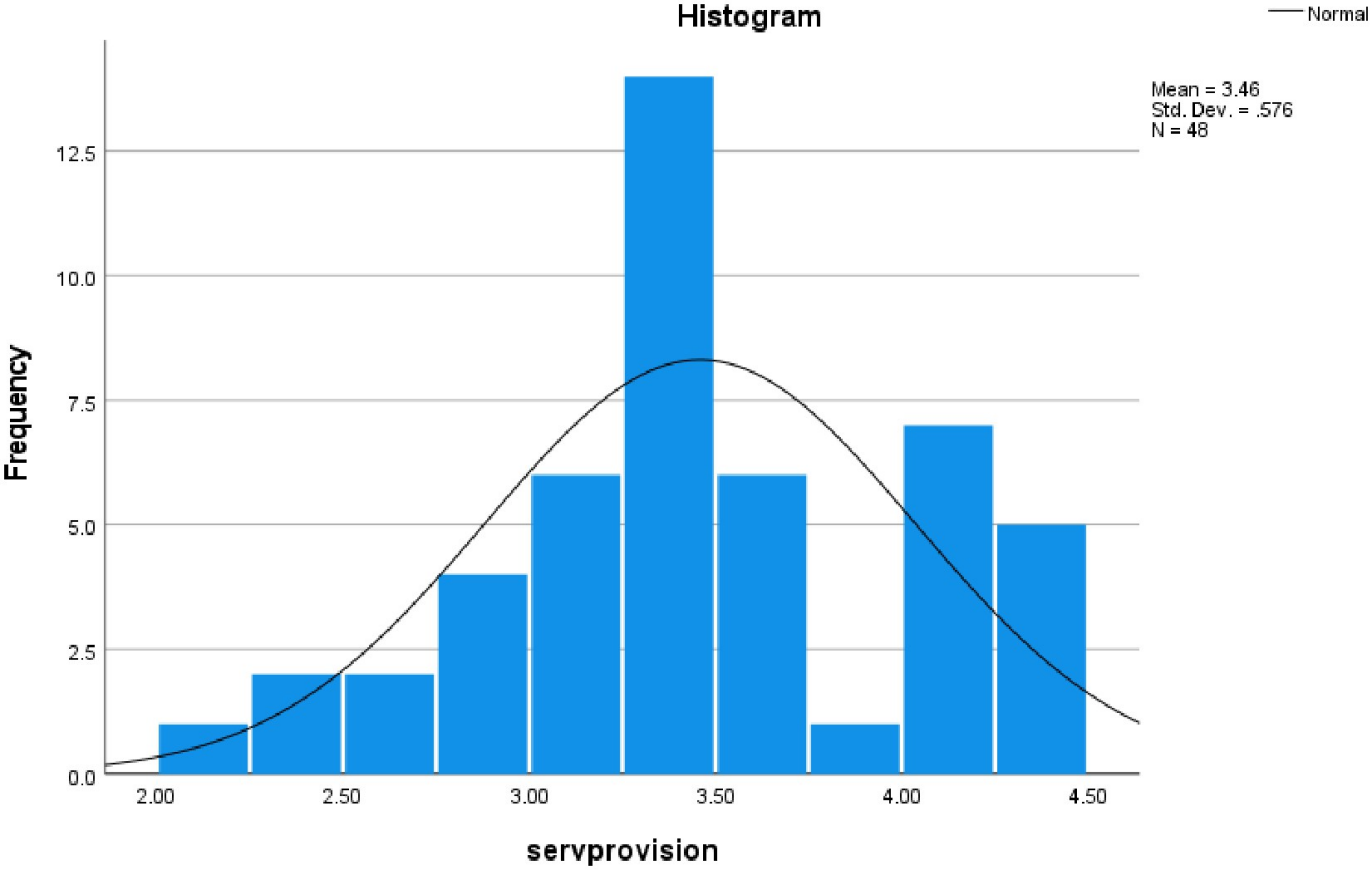
Distribution of the extent of community pharmacists’ involvement mean scores (Shapiro–Wilk *p*-value = 0.181).

The extent of community pharmacists’ involvement in preventing and controlling CVDs is presented in Table 2. Fig 2 presents the frequency distribution curve. The reliability of the scale was high, with Cronbach’s alpha = 884, n = 19).

**Table 2:** Extent of community pharmacists’ involvement in preventing and controlling CVDs and cost of service (n = 48).

Community pharmacists’ services to CVD patients (Cronbach’s Alpha = 0.884, n = 19, Shapiro–Wilk *p* = value = 0.181)	Category	Never(%)	Rarely(%)	Sometimes(%)	Often(%)	Always(%)	Mean (±SD)	Level of involvement	Cost of service (Maluti)			
									Median(Min-Max)	Q1	Q3	IQR
Dispensing prescription-only medicine	Dispensing	0	1(2.1)	6(12.5)	17 (35.4)	28(50.0)	4.33(±.781)	High	N/A			
Dispensing over-the-counter medicine (e.g. supplements)		0	0	3(6.3)	11(22.9)	34 (70.8)	4.65(±.60)	High	N/A			
Screening for Hypertension	Screening	0	1(2.1)	4(8.3)	19(39.6)	24(50.0)	4.38(±.73)	High	5.00(0.00–50.00)	5.00	10.00	5.00
Screening for Diabetes		1(2.1)	0	10(20.8)	16(33.3)	21(43.8)	4.17(±.91)	High	15.00(0.00–100.00)	15.00	20.00	5.00
Screening for hyperlipidemia (cholesterol)		30(62.5)	6(12.5)	5(10.4)	6(12.5)	1(±2.1)	1.79(±1.18)	Low	47.50(0.00–130.00)	10.00	50.00	40.00
Screening for undiagnosed CVDs (heart attacks and strokes)		11(22.9)	16(33.3)	15(31.3)	6(12.5)	0(±0.0)	2.33(±.98)	Low	0.00(0.00–75.00)	0.00	10.00	10.00
Alcohol abuse management	Lifestyle counselling	11(22.9)	12(25.0)	15(31.3)	7(14.6)	3(6.3)	2.56(±1.18)	Low	0.00(0.00–300.00)	0.00	0.00	0.00
Smoking cessation advice		3(6.3)	6(12.5)	18(37.5)	13(27.1)	8(16.7)	3.35(±1.10)	Low	0.00(0.00–200.00)	0.00	0.00	0.00
Weight management advice		1(2.1)	2(4.2)	11(22.9)	25(52.1)	9(18.8)	3.81(±.87)	High	0.00 (0.00–100.00)	0.00	2.00	2.00
Patient self-care advice (Personal testing Record) (n = 47)	Self-care management	3(6.4)	6(12.8)	14(29.8)	16(34.0)	8(17.0)	3.43(±1.12)	Low	0.00(0.00–50.00)	0.00	0.00	0.00
CVD education (n = 46)		0	2(4.3)	11(23.9)	21(45.7)	12 (26.1)	3.93(±.83)	High	0.00(0.00–250.00)	0.00	0.00	0.00
Disseminate disease information to patients (e.g. pamphlets (n = 47)		15(31.9)	13(28.0)	10(21.3)	6(12.8)	3(6.3)	2.34(±1.24)	Low	0.00(0.00–250.00)	0.00	0.00	0.00
Medication use review/Drug utilisation review (n = 47)	Medication management therapy	0	5 (10.6)	15(31.9)	14(29.8)	13(27.7)	3.74(±.99)	High	0.00(0.00–50.00)	0.00	0.00	0.00
Identify drug-related problems (n = 47)		0	3(6.4)	14(29.8)	19(40.4)	11(23.4)	3.81(±.88)	High	0.00(0.00–60.00)	0.00	0.00	0.00
Inter-professional communication regarding the patient health (n = 47)	Inter-professional collaboration	4(8.5)	7(14.9)	14(29.8)	11(23.4)	11(23.4)	3.38(±1.24)	Low	0.00(0.00)	0.00	0.00	0.00
Referral of the patient (n = 47)		0	5(10.6)	16(34.0)	14(29.8)	12(25.5)	3.70(±.98)	High	0.00(0.00–10.00)	0.00	0.00	0.00
Follow-up on patients (n = 47)	Other services	1(2.1)	2(4.3)	11(23.4)	21(44.7)	12(25.5)	3.87(±.92)	High	0.00(0.00–50.00)	0.00	0.00	0.00
Make appointments with patients (n = 46)		3(6.5)	7(15.2)	13(28.3)	14(30.4)	9(19.6)	3.41(±1.17)	Low	0.00(0.00–100.00)	0.00	0.00	0.00
Keeping patient medical record database		11(22.9)	13(27.1)	8(16.7)	9(18.8)	7(14.6)	2.75(±1.39)	Low	0.00(0.00)	0.00	0.00	0.00
**Weighted mean average**							3.46(±.58)		-	-	-	-

CVDs: Cardiovascular diseases, SD: Standard deviation, Q: Quartile, IQR: Interquartile range, N/A: Not applicable, Min: Minimum value, Max: Maximum value, Likert scale: Never = 1, rarely = 2, sometimes = 3, often = 4, always.

### Dispensing

Above 80% of the respondents were either often involved or always involved in dispensing prescription-only and over-the-counter medicine (mean = 4.33±.78 and 4.65±.60, respectively). There were significant differences (*t*(46) = 3.065, *p* = 0.004 and *t*(46) = 2.615, *p* = 0.012) in the mean scores between community pharmacists who had a weight scale and blood glucose testing device (mean = 4.44±.70 and 4.39±.71) and those who did not have the devices (mean = 3.40±.89 and 3.00±1.41) respectively.

### Disease screening

Hypertension and diabetes screening were the most common (mean = 4.38±.73 and 4.17±.91, respectively) screening services. Community pharmacists operating from Maseru (mean = 4.34±0.91) were more motivated to perform diabetes screening than those from other districts (mean = 3.69±.75); (*t*(46) = 2.308, *p* = 0.001). The mean scores for the screening of hypertension differed between those who possessed and did not possess a weight scale and a blood glucose testing device (*t*(46) = 3.498, *p* = 0.001 and *t*(46) = 4.341, *p*<0.001, respectively). Most respondents (62.5%) had never performed cholesterol screening tests, while 56.2% rarely or had never screened patients for undiagnosed CVDs. There were significant differences in the provision of a cholesterol screening and the location of a pharmacy, the number of CVD patients seen daily, the availability of a cholesterol testing device, and the availability of standard treatment guidelines in a pharmacy. Firstly, there was a significant difference between community pharmacists working in urban areas (mean = 2.24±1.45) and those in the suburban and rural areas (mean = 1.44±.80); (*t*(29) = 2.260, *p* = 0.031). Secondly, community pharmacists seeing more CVD patients daily (mean = 2.30±1.42) differed significantly from those seeing fewer patients (mean = 1.43±.84); (*t*(28) = -2.460, *p* = 0.020). Similarly, the availability of a cholesterol testing device (mean = 3.06±1.06) and the standard treatment guidelines (mean = 1.84±1.21) differed significantly compared to groups with no devices (mean = 1.16±.57) and guidelines (mean = 1.00±.00); *t*(19) = 6.703, *p*<0.001 and *t*(44) = 4.700, *p*<0.001 respectively.

#### Lifestyle counselling

The most provided lifestyle counselling services was weight management (mean = 3.81±.87). There was a significant difference (*t*(45) = 2.114, *p* = 0.040) between community pharmacists who were owners of the pharmacy (mean = 4.11±0.83) and those who were employees (mean = 3.59±0.83). Alcohol abuse counselling was the least common service with a significant difference between more experienced (mean = 3.06±1.06) and less experienced (mean = 2.31±1.18) community pharmacists; *t*(46) = -2.148, *p* = 0.045.

#### Self-care management

Over 70% (mean = 3.93±.83) of the respondents were often to always involved in CVD education to patients. There was no significant mean difference between independent groups (*p*>0.05). On the other hand, fewer community pharmacists (mean = 2.34±1.24) were involved in information dissemination to patients through pamphlets. There was a significant difference in the dissemination of information through pamphlets between more experienced community pharmacists (mean = 2.87±1.19) and those less experienced (mean = 2.09±1.20); *t*(46) = -2.148, *p* = 0.037. Correspondingly, a modest significant difference was observed between community pharmacists located in the Maseru district (mean = 2.56±1.21) and those located in other districts (mean = 1.77±1.17); *t*(45) = 2.020, *p* = 0.049.

#### Medication management therapy

Most respondents were often to always involved in medication use review (57.5%) and identification of drug-related problems (63.8%). There was a significant difference in the identification of drug-related problems between community pharmacists who had the standard treatment guidelines (mean = 3.89±0.84) and those without the guidelines in their pharmacies (mean = 2.67±.58); *t*(45) = 2.459, *p* = 0.018.

#### Inter-professional collaboration

Patient referral was the most common inter-professional collaboration practice compared with inter-professional communication. All respondents were involved in patient referral at varying levels (mean = 3.70±.98). Community pharmacists who possessed a blood glucose testing device (mean = 3.78±.93) compared to those who did not possess the device (mean = 2.00±.00) demonstrated significantly higher involvement in patient referral; *t*(44) = 12.876, *p*<0.001.

#### Other services

The pharmacists mostly (70.2%) often or always follow up with their patients (mean = 3.87). Similarly, the majority of the respondents showed varying levels (mean = 3.41) of involvement in scheduling appointments with their patients, with 3 out of 46 (6.5%) never providing this service. On the other hand, 50% of respondents (n = 46) showed rarely to no involvement in keeping patient medical databases.

*Community pharmacists’ attitudes and perceptions*. Community pharmacists’ attitudes and perceptions of their role in preventing and controlling CVDs are presented in Table 3. The constructs were classified according to the TPB constructs: attitude, subjective norms, and perceived behavioural.

**Table 3:** Attitudes and perceptions of community pharmacists towards their role in preventing and controlling CVDs (n = 48).

Attitudes and perceptions	Responses						ANOVA *(p* = value)
	Strongly disagree(%)	Disagree(%)	Neutral(%)	Agree(%)	Strongly agree(%)	Mean(±SD)	
**Attitude (Cronbach’s Alpha = 0.719, Shapiro-Wilk *p* = 0.039) mean = 4.46(±.39))**							
If I provide health promotion services (such as lifestyle modification) to HTN patients, I feel that I am doing something positive for the patients	0	0	0	17(35.4)	31(64.6)	4.65(±48)	0.088
Patient self-monitoring of CVD risk factors forms part of community pharmacist services	1(2.1)	2(4.2)	10(20.8)	24(50.0)	10(20.8)	3.85(±.88)	0.088
Involvement of a community pharmacist in the primary healthcare framework in the prevention and control of CVDs can result in satisfactory disease outcome	0	0	1(2.1)	8(16.7)	39(81.3)	4.79(±.46)	0.088
Integrating CVD health promotion into community pharmacy daily practice is important	0	0	4(8.3)	19(39.6)	25(52.1)	4.44(±.65)	0.088
CVD prevention and control should form part of community pharmacy practice standards in Lesotho	0	0	0	15(31.3)	33(68.8)	4.69(±.47)	0.088
CVD health promotion is part of community pharmacy practice standard	0	0	3(6.3)	26(54.2)	19(39.6)	4.33(±.60)	0.088
**Subjective norms (Cronbach’s Alpha = 0.641)**							
Physicians believe that I should advise HTN patients on lifestyle modification	0	6(±12.8)	22(46.8)	15(31.9)	4(8.5)	3.36(±.82)	0.643
It is my employment obligation that I provide HTN patients with lifestyle modification	0	1(2.2	6(13.0)	14(30.4)	25(54.3)	4.37(±.80)	0.532
HTN Patients appreciate the pharmacist’s effort to counsel them]	1(2.1)	1(2.1)	9(18.8)	25(52.1)	12(25.0)	3.96(±.85)	0.886
**Perceived behavioral control (Cronbach’s Alpha = 0.569)**							
I am confident that I can counsel HTN patients on lifestyle modification	0	2(4.2)	1(2.1)	12(25.0)	33(68.8)	4.58(±.74)	0.123
Providing lifestyle modification counselling to HTN patients is easy	1(2.1)	7(14.6)	8(16.7)	18(37.5)	14(29.2)	3.77(±1.10)	0.078
I am able to initiate lifestyle counselling on my own decision	0	3(6.3)	3(6.3)	23(47.9)	19(39.6)	4.21(±.82)	0.001*

CVDs: Cardiovascular diseases, HTN: Hypertension, Likert scale: Strongly disagree = 1, disagree = 2, neutral = 3, agree = 4, strongly agree = 5,

* = statistical difference.

The attitude scale had a reliability of 0.719. Therefore, all the constructs were analysed as one composite variable. A Shapiro-Wilk test showed a *p* = 0.039, indicating an asymmetric data distribution. Consequently, a non-parametric Mann Whitney U test was used. The respondents showed a unanimous positive attitude (mean = 4.46(±.39) to questions such as "if I provide health promotion services (such as lifestyle modification) to hypertension patients, I feel that I am doing something positive to the patients" (Table 3). The mean scores for the attitude significantly differed between community pharmacies in Maseru (mean rank = 21.49) and those in other districts (mean rank = 32.62); Z = -2.467, *p* = 0.014. All constructs showed no significant association with the extent of community pharmacists’ involvement in preventing and controlling CVDs, *p*>0.05.

The subjective norms and perceived behavioural control scales had reliability values of 0.641 and 0.569, respectively. Thus, individual constructs were analysed separately. Similarly to attitude, respondents demonstrated a unanimous positive perception (>3) of all constructs, such as "hypertension patients appreciate the pharmacist’s effort to counsel them (subjective norms)" and "I am confident that I can counsel hypertension patients on lifestyle modification (perceived behavioural control)". There were significant differences in the construct "hypertension patients appreciate community pharmacists’ effort to counsel them" between gender and employment status independent groups. Males (mean = 4.21±.74) than females (3.60±.88) perceived patients as appreciative of their counselling services; *t*(46) = -2.335, *p* = 0.024. Similarly, there was a significant difference between community pharmacists who were pharmacy owners (mean = 4.39±.61) and those who were employees (mean = 3.66±.86); *t*(45) = 3.167, *p* = 0.003. Compared with other constructs, a modest agreement was observed for the subjective norms "physicians believe that I should advise hypertension patients on lifestyle modification", with less than half of the respondents (40.4%) demonstrating agreement. There were no significant differences between independent groups. In the same way, no significant difference observed in the extent of community pharmacists’ involvement between community pharmacists who agreed and disagreed with the constructs in the subjective norms, *p*>0.05.

The perceived behavioural control construct "I am confident that I can counsel hypertension patients on lifestyle modification" significantly differed between pharmacies with ≥2 community pharmacists (mean = 4.85±.38) and pharmacies with 1 pharmacist (4.49±.82); *t* (43) = -2.082, *p* = 0.043. There was a significant difference in the extent of community pharmacists’ involvement between independent groups of the construct "I am able to initiate lifestyle counselling on my own decision", *p* = 0.001.

### Barriers and facilitators

The barriers and facilitators were assessed against 22 and 8 constructs, respectively (Table 4). The barrier constructs were further categorised into four factors: community pharmacy/pharmacist, patient, inter-professional relations, and regulatory aspects.

**Table 4:** Barriers and facilitators in the provision of services to CVD patients in Lesotho (n = 48).

Scale item	Category	Strongly disagree(%)	Disagree(%)	Neither agree nor disagree(%)	Agree(%)	Strongly agree(%)	Mean (SD)	Category
**Barriers (Cronbach’s Alpha = 0.754, n = 16, Shapiro-Wilk *p* = 0.219, mean = 3.11±.48)**								
HTN patients’ inability to obey medication adherence instructions leads to unfavourable outcomes	Patient	1(2.1)	0(0)	2(4.2)	27(56.3)	18(37.5)	4.27(±.74)	Most common
Patients’ lack of awareness of community pharmacists’ services is a barrier to the provision of services to patients	Patient	2(4.2)	5(10.4)	2(4.2)	22(45.8)	17(35.4)	4.16(±.95)	Most common
Lack of time by the patient is a barrier to the provision of services to patients	Patient	1(2.1)	3(6.3)	5(10.4)	23(47.9)	16(33.3)	3.98(±.98)	Most common
Lack of interest by HTN patients is a barrier to the provision of services to HTN patients	Patient	1(2.1)	7(14.6)	4(8.3)	23(47.9)	13(27.1)	3.83(±1.06)	Most common
Lack of communication between community pharmacists and other healthcare providers is a barrier to the provision of care to HTN patients	Professional relations	2(4.2)	4(8.3)	9(18.8)	20(41.7)	13(27.1)	3.79(±1.07)	Most common
Lack of integration between community pharmacy-based services and other healthcare providers is a barrier to the provision of health promotion services to HTN patients	Regulatory	1(2.1)	7(14.6)	10(20.8)	18(37.5)	12(25.0)	3.69(±1.06)	Most common
Lack of personnel and tools/equipment is a barrier to the provision of services to patients	Community pharmacy/pharmacist	4(8.3)	6(12.5)	4(8.3)	22(45.8)	12(25.0)	3.64(±1.22)	Most common
The socio-economic status of patients discourages HTN patients from satisfactorily adhering to their medication	Patient	3(6.3)	6(12.5)	7(14.6)	22(45.8)	10(14.6	3.63(±1.14)	Most common
It is difficult to get people to avoid CVD risk behaviours/to engage in healthy lifestyles	Community pharmacy/pharmacist	2(4.2)	11(22.9)	8(16.7)	17(35.4)	10(20.8	3.46(±1.18)	Most common
Fragmented supply chain regulations hinder my full potential to provide healthcare services to HTN patients	Regulatory	2(4.2)	9(18.8)	9(18.8)	22(45.8)	6(12.5)	3.44(±1.10)	Most common
Lack of financial incentives discourages me from providing health promotion services to HTN patients	Community pharmacy/pharmacist	9(18.8)	17(35.4)	6(12.5)	10(20.8)	6(12.5)	2.73(±1.33)	Less common
I feel uncomfortable asking patients about their CVD risk factors, e.g. smoking.	Community pharmacy/pharmacist	20(41.7)	17(35.4)	5(10.4)	1(2.1)	5(10.4)	2.00(±1.26)	Less common
I believe I do not have adequate knowledge and skills in CVD management	Community pharmacy/pharmacist	21(43.8)	16(33.3)	7(14.6)	4(8.3)	0(0)	1.88(±.96)	Less common
I am not interested in providing health promotion services to HTN patients	Community pharmacy/pharmacist	28(58.3)	15(31.3)	2(4.2)	1(2.1)	2(4.2)	1.61(±1.00)	Less common
Lack of electricity at my pharmacy location forces me to knock off earlier than would be preferred]	Community pharmacy/pharmacist	23(47.9)	16(33.3)	5(10.4)	2(4.2)	2(4.2)	1.60(±1.19)	Less common
Health promotion services to HTN patients are not the role of community pharmacists	Community pharmacy/pharmacist	30(62.5)	16(33.3)	1(2.1)	0(0)	1(2.1)	1.46(±.74)	Less common
**Facilitators (Cronbach’s Alpha = 0.823, n = 7, Shapiro-Wilk *p* = 0.144, mean = 3.60±.75)**								
My relationship with other community pharmacists is good	Professional relations	0(0.0)	1(2.1)	5(10.4)	21(43.8)	21(43.8)	4.62(±.80)	Most common
I have enough time to provide medication adherence counselling to every single HTN patient	Community pharmacy/pharmacist	2(4.2)	14(29.2)	2(4.2)	22(45.8)	8(16.7)	3.48(±1.15)	Less common
My relationship with physicians involved in cardiovascular care motivates me to provide medication counselling and weight loss advice to HTN patients	Professional relations	1(2.1)	10(20.8)	11(22.9)	17(35.4)	9(18.8)	3.48(±1.10)	Less common
HTN patients are always willing to receive my medication and weight advise		0(0)	5 (10.4)	5 (10.4)	30 (62.5)	8 (16.7)	3.85 (±.83)	Most common
There is enough support from the regulatory authority (Ministry of Health) that encourages my provision of medication adherence counselling to HTN patients	Regulatory	15(31.3)	19(39.6)	6(12.5)	4(8.3)	4(8.3)	2.08(±1.52)	Less common
My pharmacy has a sufficient number of staff to provide medication adherence counselling to HTN patients	Community pharmacy/pharmacist	4(8.3)	11(22.9)	10(20.8)	13(27.1)	10(20.8)	3.29(±1.27)	Less common
A private area is available at my pharmacy to provide counselling	Community pharmacy/pharmacist	3(6.3)	2(4.2)	0(0)	18(37.5)	25(52.1)	4.25(±1.10)	Most common

CVDs: Cardiovascular diseases, SD: Standard deviation, HTN: Hypertension, Likert scale: Strongly disagree = 1, disagree = 2, neutral = 3, agree = 4, strongly agree = 5.

#### Community pharmacy/Pharmacist factors

Limited human resources and equipment (mean = 3.64±1.22) and difficulty getting people to adopt healthy lifestyles (mean = 3.46±1.18) were the most common barriers, with 70.8% and 56.2% of respondents agreeing to strongly agreeing, respectively. There was a modest significant difference (*t*(24) = 2.05, *p* = 0.049) in difficulty getting people to adopt healthy lifestyles between respondents with <5 years and ≥5 years of community pharmacy experience.

#### Patient factors

Most of the respondents (>75%) agree to strongly agree that lack of medication adherence (mean = 4.27±.74), lack of awareness of community pharmacist services (mean = 4.16±.95), lack of time (mean = 3.98±.98), and lack of interest (mean = 3.83±1.06) by patients hindered the successful provision of health promotion towards CVD management. There was no significant difference in patient-factor barriers between independent groups (*p*>0.05).

#### Inter-professional relations

A lack of communication between community pharmacists and other healthcare providers (mean = 3.79±1.07) was identified as a major barrier to the provision of health promotion. Most respondents (68.8%) agree to strongly agree that there was a lack of inter-professional communication. There were no significant differences in inter-professional barriers between independent groups (*p*>0.05).

#### Regulatory factors

Most community pharmacists (70.9%) disagreed to strongly disagreed that there was enough support from the regulatory authorities to encourage their provision of health promotion services to CVD patients, with a significant difference (*t*(17) = -2.22, *p* = 0.040) between pharmacies with other healthcare professional employees (mean = 2.69±1.80) and those with no other healthcare professionals besides pharmacists (mean = 1.46±.88). Correspondingly, the majority of the respondents agreed to strongly agreed that lack of integration between community pharmacy-based services and other healthcare providers (62.5%) and disjointed supply chain regulations (58.3%) hindered their service provision in the management of CVDs. There were no significant mean differences in lack of integration and disjointed supply chain regulations between independent groups (*p*>0.05).

#### Recommendations for enhancing community pharmacists’ services

**Fig 3:**
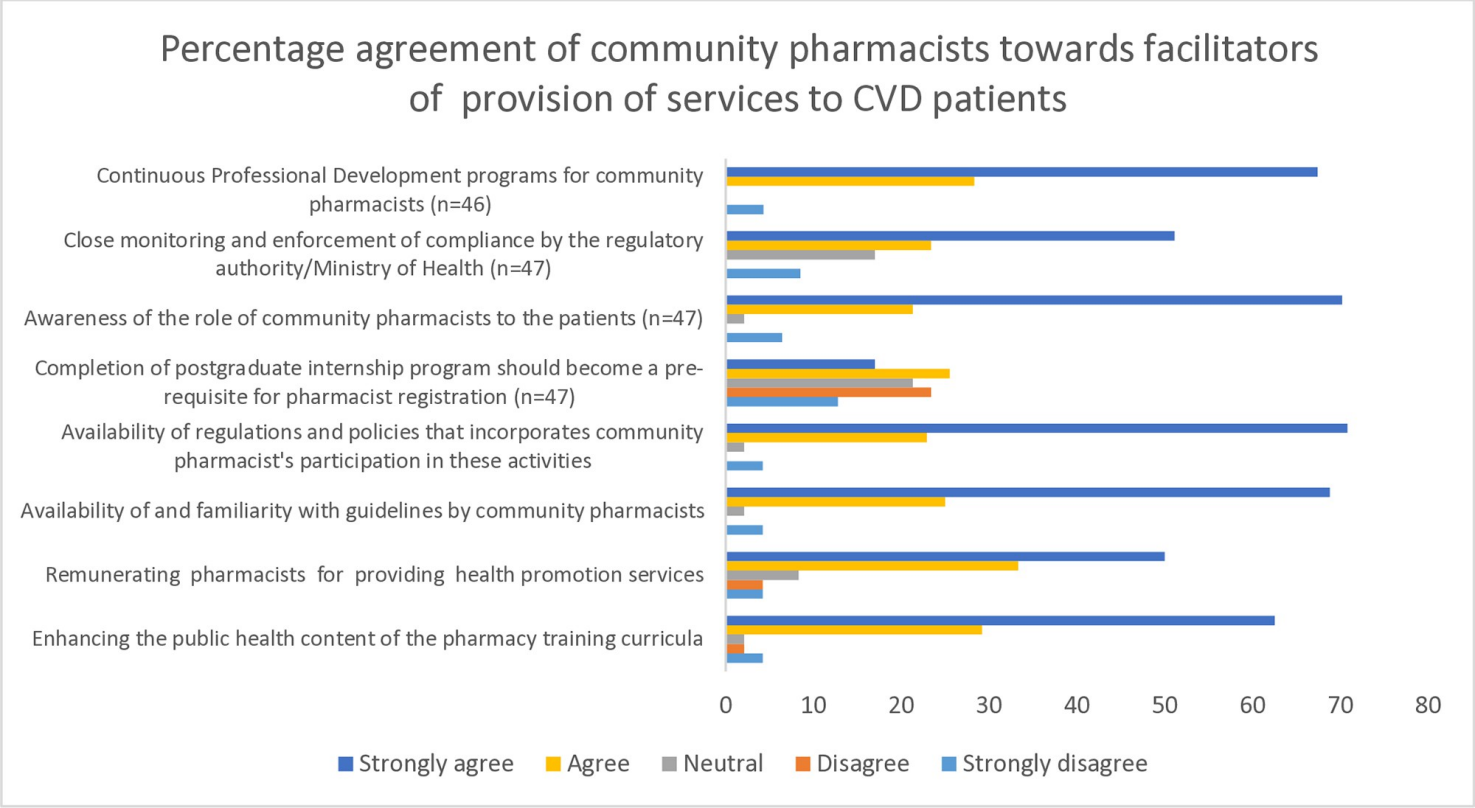
Recommendations for enhancing community pharmacists’ provision of services to CVD patients.

Most respondents (>70%) agreed to strongly agreed to 7 out 8 constructs as roles needed for the enhancement of community pharmacy services to CVD patients (Fig 3). On the other hand, 42.5% of respondents agreed that a postgraduate internship program should be a prerequisite for pharmacist registration, while 36.2% disagreed. There was no significant differences between independent groups (*p*>0.05).

### Tools availability

Table 5 presents the availability of testing devices for blood pressure, blood sugar, cholesterol, body weight, and Lesotho’s standard treatment guidelines. All community pharmacies (n = 48) had blood pressure testing devices, while cholesterol measuring devices were the least common (33.3%). There were significant differences in the availability of a measuring tape, height measuring device, body weight scale, and blood cholesterol measuring device between independent groups in Table 1. Firstly, the availability of a measuring tape was significantly associated with experience (X^2^ = 6.095, df = 1, *p* = 0.014). A modest difference between age groups (X^2^ = 3.884, df = 1, *p* = 0.049) was observed. Secondly, the availability of a height measuring device was significantly associated with the district where the pharmacy was located (X^2^ = 4.704, df = 1, *p* = 0.030). And thirdly, the number of CVD patients seen daily was associated with the availability of body weight scale (X^2^ = 3.981, df = 1, *p* = 0.046) and blood cholesterol measuring device (X^2^ = 4.286, df = 1, *p* = 0.038).

**Table 5:** Availability of tools in the community pharmacies (n = 48).

CVD tools	Availability			
	Yes		No	
	Frequency	Percentage (%)	Frequency	Percentage
Blood glucose measuring device (Glucometer)	46	95.8	2	4.2
Blood pressure measuring device	48	100	0	0
Measuring tape	27	56.3	21	43.7
Height measuring device	27	56.3	21	43.7
Body weight scale/balance	43	89.6	5	10.4
Blood cholesterol measuring device	16	33.3	32	66.7
Standard treatment guidelines for Lesotho	45	93.7	3	6.3

CVD: Cardiovascular disease.

### Knowledge of CVDs risk factors by community pharmacists

Table 6 presents the results of community pharmacists’ knowledge of CVD risk factors. The majority of the respondents demonstrated good knowledge in 19 of 23 constructs. Respondents showed moderate knowledge of cholesterol (54.2%) and diabetes-related (64.6%) risk factors and low knowledge (27.1%) of gender and other cholesterol-related risk factors, (*p*>0.05). Overall, an average knowledge score of 85.3% was achieved, demonstrating good knowledge of CVD risk factors.

**Table 6:** HDQF item with correct responses, proportion, and corrected item-total correlation (n = 48).

HDQF Item	Correct response	No. of correct responses	Valid % correct responses
A person is always aware when they have CVDs	False	40	83.3
Hyperlipidaemia (high cholesterol) increases the chances of CVDs	True	46	95.9
The risk of CVDs increases with age	True	48	100
Controlled blood pressure will reduce a person’s risk of CVD progression	True	48	100
A diabetic patient can reduce his/her risk of CVDs if his/her cholesterol level is controlled	True	47	97.9
Diabetic patients often have low HDL cholesterol	True	18	37.5
Regular physical exercise will lower the risk of CVDs	True	46	95.9
Overweight and obesity are risk factors for CVDs	True	47	97.9
A fatty meal has no effects on blood cholesterol levels	False	46	95.9
A person who smokes is at risk of CVDs (n = 47)	True	44	93.6
Diabetes is a risk factor for CVDs (n = 45)	True	43	95.6
High blood sugar makes the heart work harder	True	31	64.6
Hypertension is a risk factor for CVDs (n = 46)	True	46	100
Diabetic patients hardly have high cholesterol	False	36	75
A family history of heart disease increases the risk of heart disease (n = 45)	True	43	95.6
Gender is a risk factor for CVD progression in patients with diabetes	False	13	27.1
A person whose HDL is high is at risk for CVDs	False	26	54.2
Prolonged increase in blood sugar levels can trigger an increase in cholesterol and increased chances of CVDs	True	38	79.2
A diabetic patient can reduce his/her risk of getting CVDs if his/her weight is kept under control (n = 46)	True	45	97.8
A person whose LDL is high is at risk of CVDs (n = 45)	True	38	84.4
A diabetic patient can reduce his/her chances of getting CVDs if his/her sugar blood levels are kept under control (n = 46)	True	46	100
A diabetic patient can reduce his/her chances of getting CVDs if his/her blood pressure level is kept under control (n = 45)	True	45	100
Smoking cessation lowers risk of developing CVDs (n = 47)	True	44	93.6
Physical exercise will lower a person’s chances of CVDs if it is taken only at a gym or coached by an instructor (n = 46)	False	42	91.3
Walking and gardening are not considered exercise that will help lower the risk of developing CVDs	False	37	77.1
**Average %**			**85.3**

CVDs: Cardiovascular diseases, SD: Standard deviation, HDFQ: Heart disease fact questionnaire, HDL: High-density lipoprotein, LDL: Low-density lipoprotein.

### Additional services

Table 7 displays the results of extra services community pharmacists are willing to provide in the future. The majority of the respondents believed that they needed to do more for CVD patients. More than 85% of community pharmacists agreed that they should broaden disease education coverage, create social groups to encourage lifestyle modification, increase screening capacity with advanced testing devices, and monitor patients’ treatment. Approximately half (52.1% agreement) of the respondents wanted to see diagnosing and prescribing at a fee added to the community pharmacists’ services to CVD patients.

**Table 7:** Additional services in preventing and controlling CVDs (n = 48).

Additional service	Strongly disagree (%)	Disagree	Neutral	Agree	Strongly agree	Mean (SD)
What we are currently doing is enough	12 (25.0)	26 (54.2)	8 (16.7)	1 (2.1)	1 (2.1)	2.02 (±.838)
Utilise various platforms (e.g. pamphlets, social media, radios, TV) to educate the public about CVDs	2 (4.2)	0	0	10 (20.8)	36 (75.0)	4.63 (±.866)
Diagnose and prescribe at a fee	1 (2.1)	9 (18.8)	13 (27.1)	18 (37.5)	7 (14.6)	3.44 (±1.029)
Create patient groups to encourage lifestyle modification	2 (4.2)	1 (2.1)	3 (6.3)	25 (52.1)	17 (35.4)	4.13 (±.937)
Community outreach services	2 (4.2)	0 (0)	4 (8.3)	14 (29.2)	28 (58.3)	4.38 (±.959)
Increase screening capacity services	2(4.2)	0	1 (2.1)	20 (41.7)	25 (52.1)	4.35 (±.934)
Monitoring and review of treatment]	2(4.2)	0	0	16(33.3)	30(62.5)	4.50 (±.875)
Use of advanced testing devices (n = 47)	2 (4.3)	0	3 (6.4)	24 (51.1)	18 (38.3)	4.19 (±.900)

CVDs: Cardiovascular diseases, SD: Standard deviation, TV: The television set.

## Discussion

Community pharmacists are easily accessible health professionals and are well positioned to contribute to the primary healthcare of CVDs. The role of community pharmacists in preventing and controlling CVDs has been studied mainly in HICs, leaving a gap in knowledge about their roles in LMICs [36]. The study presents a quantitative analysis of community pharmacists’ extent of involvement in preventing and controlling CVDs in Lesotho and factors associated with such involvement. To the best of the authors’ knowledge, this is the first study that provides a comprehensive evaluation of community pharmacists’ level of involvement, attitude, and perceptions in preventing and controlling CVDs in Lesotho. Previous qualitative study conducted by the authors revealed that community pharmacists offered a range of services including traditional medication dispensing and health promotion activities such as screening tests, lifestyle counselling, adherence counselling, and disease education [17]. The current study adds a quantitative analysis of community pharmacists’ level of involvement, attitudes, perceptions, and influence of independent variables in the prevention and control of CVDs.

### Involvement of community pharmacists in preventing and controlling CVDs

Apart from dispensing, the most common health promotion activities were hypertension and diabetes screening, weight management services, CVD education, medication management therapy, and referral of and follow-up on patients. This is an important observation considering the increasing prevalence of diabetes and hypertension over the years and the risk they pose towards the development of CVDs [5, 6]. However, the involvement of community pharmacists in cholesterol and undiagnosed CVDs screening was relatively low and needed to be improved. Similar findings were reported in Ghana, where community pharmacists were highly involved in disease screening, lifestyle counselling, and medication management therapy services [37]. Regarding lifestyle modification, community pharmacists were highly involved in weight management counselling. The availability of a weight scale (89.6%) supports the involvement of community pharmacists in weight management activities. However, almost half of the respondents did not have a measuring tape or a height measuring device, which are essential for waist circumference and body mass index measurements [38]. The findings underscore the need for standardisation of community pharmacy practice services.

On the other hand, smoking cessation and alcohol abuse advice were less practised compared to weight management counselling. The results contradict the findings of Sendekie and Netere [21] in Ethiopia, in which pharmacists were highly involved in smoking and alcohol abuse counselling [21]. The disparities could be due to a lack of competency. For instance, community pharmacists with more than 5 years of experience were more involved in alcohol abuse counselling than those with less than 5 years in the current study. This gap can be improved by developing continuous professional development strategies for community pharmacists which is currently lacking. Generally, the study shares similarities with previous findings and shows community pharmacists’ involvement in medicine dispensing, disease screening, lifestyle counselling, and health education in CVDs [20, 31, 39]. Future studies should focus on the effectiveness of these services in reducing CVD risk.

### Knowledge of CVD risk factors

Overall, community pharmacists demonstrated good knowledge of CVD risk factors. There is need for continuous professional development to improve knowledge mainly on diabetes and cholesterol-related risk. While all the respondents admitted to receiving CVD management education during their pharmacy training, the majority (83.3%) had never received related postgraduate training. A life-long commitment to learning is crucial in pharmaceutical care since acquiring sufficient pharmaceutical skills and competencies at school is impossible [40]. Thus, community pharmacies in Lesotho must create platforms that support and motivate continuous professional development for community pharmacists. Our results are consistent with findings in Nigeria and Saudi Arabia, where community pharmacists showed good knowledge of CVD risk factors with modest knowledge of cholesterol and diabetes [30, 41].

In contrast, the knowledge score was comparatively higher than that of community pharmacists in Ethiopia (55.6%) [42]. The difference could be awareness of guidelines due to the availability of standard treatment guidelines in most community pharmacies in Lesotho (93.7%) compared with a small proportion of community pharmacists who were aware of national guidelines for CVDs in Ethiopia [42].

### Barriers and facilitators

Effective community pharmacists’ health promotion services have been reported in preventing and controlling CVDs [12, 43]. However, community pharmacy practice is surrounded by factors that discourage their scope of practice globally [31, 44, 45]. Lesotho is no exception to global concerns since community pharmacists experience barriers that cut across pharmacy, patient, inter-professional relations, and regulatory factors. The topmost reported barriers were patient factors such as non-adherence to medication, unawareness of community pharmacist services, and lack of time and interest. Consequently, respondents believed that patients’ awareness of community pharmacists’ services should be enhanced.

On the other hand, difficulty getting patients to adopt healthy lifestyles differed significantly between most and less experienced community pharmacists. The findings underscore the need for continuous professional development for pharmacists to encourage standardised provision of services to patients. The enactment of the Lesotho Medicines and Medical Devices Control Authority Act 2023 in August 2023 brings hope to pharmacy practice in Lesotho and possible regulatory changes that can drive service improvement among community pharmacists [46]. The study findings share several similarities with results from LMICs. In Ethiopia, the most reported barriers to CVD health promotion were the unavailability of equipment, lack of financial motivation, and lack of patient acceptance of pharmacists’ services, and the percentage agreement ranged between 64% and 86% [42]. Additionally, Rwanda’s most perceived barriers were poor inter-professional collaboration, unavailability of equipment, and lack of standard guidelines (agreement of 57%-69%) [31]. On the other hand, in Saudi Arabia, the most reported barriers were lack of time (66% agreement), followed by educational resources (41%) and lack of confidence in using devices (36%) [44]. Likewise, Qatar pharmacists perceived a lack of time (53%), a shortage of human resources (42%), and a lack of private areas for counselling [47] as barriers’ to community pharmacists’ service provision. Alavudeen, Easwaran [44] and El Hajj, Abu Yousef [47] suggest that pharmacy/community pharmacist factors influence the most important barriers in HICs, while our findings point to patient factors. Future research should explore the perceptions of patients, other healthcare professionals involved in CVD care, and regulatory authorities regarding the role of community pharmacists in CVD management in Lesotho.

### Attitudes and perceptions

Respondents’ attitudes and perceptions were assessed using three TPB constructs: attitude, perceived behavioural control, and subjective norms [25]. The respondents portrayed a positive attitude in support of their role in preventing and controlling CVDs. According to Ajzen [25], attitude is a strong determinant of the intention to perform a certain behaviour [25]. The positive attitude provides the basis for the optimal utilisation of community pharmacists and integration in CVD structures in Lesotho. Furthermore, the majority of the pharmacists perceived themselves as confident in providing lifestyle modification counselling to patients with CVD risk factors. On the other hand, four out of five respondents agreed to strongly agreed that they initiate lifestyle counselling out of their own free will. The findings imply the absence of external pressures, such as regulatory or social pressures, which might subject the provision of services to a lack of consistency. Nonetheless, community pharmacists perceived patients as more appreciative of their health promotion services than physicians. To better understand the influence of social factors, future research should investigate the perceptions of patients and physicians towards community pharmacists’ provision of health promotion services to CVD patients. The findings agree with the results of studies conducted in Rwanda and Yemen [31, 48]. Similarly, Alavudeen, Easwaran [44] posited that Saudi Arabian pharmacists had a positive attitude and perceptions toward their capability to provide health promotion to CVD patients [44].

The study had limitations. Firstly, the study included reachable participants who were available to participate during the 5 months of data collection. The contact phone numbers for some community pharmacists were unreachable, while other pharmacists were not available to partake in the study. Additionally, two questionnaires were excluded from the final analysis due to their non-completion exceeding a 10% set threshold, thus further reducing the sample size below the expected. These could have affected the power of statistical tests, leading to the analysis’s inability to detect small differences between independent groups. For example, a lack of a significant difference between the respondent’s attitudes and subjective norms and the extent of involvement in CVD care could be attributed to the decreased statistical power due to a smaller sample size. Although efforts were made to achieve a higher response rate, including the use of online and paper-based questionnaires, and a relatively extended data collection period, only 50 pharmacists completed and returned the questionnaires. Furthermore, a response rate of 80.6% was comparable to previous similar studies based locally and internationally [20, 22, 49, 50]. Given the 5 month duration of the data collection, a high response rate of 80.6%, and a choice between online and paper-based questionnaires for community pharmacists, the researchers believe that the actual sample size of 50 respondents was practically feasible. Secondly, the study was conducted in four cities in the lowlands of Lesotho, with most participants being from urban areas. Therefore, the results might not be generalisable to community pharmacists in other cities and rural areas, particularly in the highlands of Lesotho. Thirdly, the cross-sectional design employed in this study presents a significant limitation, as it cannot establish the direction of the cause-and-effect relationship [51]. Furthermore, performing robust statistical analyses, such as multiple logistic regression, was not feasible due to the smaller sample size. Logistic regression typically requires a minimum of 10 to 20 events per independent variable [52] to enhance the validity of the findings; however, this study did not meet that requirement. Despite these limitations, the study evaluates Lesotho community pharmacists’ extent of involvement in preventing and controlling CVDs in Lesotho and possesses several strengths. Firstly, the participants had a comparable gender representation of 4:5 females to males and a broad coverage of community pharmacy experience ranging from six months to over 10 years. Secondly, the study represents the role of community pharmacists in preventing and controlling CVDs in different pharmacy settings, from urban and rural geographical areas to independent and chain stores. Additionally, the majority of Lesotho community pharmacies (90%) are established in the four districts studied [8] that serve an estimated population of 1.3 million people (65% of the national population) [19]. Another important attribute is that the results compare with previous findings of similar studies. Therefore, the authors are confident that the findings provide pertinent information to healthcare policy- and decision-makers, regulators in clinical practice, and future research regarding the involvement of community pharmacists in preventing and controlling CVDs. The results can inform policy development in LMICs.

## Conclusions

The provision of these CVD services differed between independent groups. Community pharmacists possess good knowledge, and positive attitudes and perceptions of their role in CVD care. Thus, they can improve CVD outcomes. However, barriers potentially limit their scope of practice and thus encourage inconsistency in providing services to the patients. Therefore, community pharmacists and pharmaceutical bodies can use the results to improve community pharmacy service delivery by facilitating processes to ensure that the identified barriers are addressed in order to maximise health outcomes for CVD patients. The policy-makers can use the study findings to integrate community pharmacists in the PHC frameworks, develop pharmacy practice guidelines that standardise community pharmacists’ provision of CVD services and enforce inter-professional collaborations. Future studies should focus on investigating the perceptions of patients, policy-makers, regulators, and other healthcare professionals regarding the role of community pharmacists in preventing and controlling CVDs in Lesotho and LMICs. The study recommends regular updating of community pharmacists’ database to ensure that the details are current. Future studies should focus other areas that are not studies such as rural areas and districts in the highlands. Moreover, future researchers should focus on establishing causal relationships between community pharmacist involvement in CVD management and independent variables by employing robust statistical models, such as logistic regression, along with random sampling techniques to enhance the validity of the findings.

## Supporting information

S1 Table Dataset. (XLSX)

S2 Table Study questionnaire. (PDF)

## References

[1] World Health Organization. *Global Atlas on cardiovascular disease prevention and control*. 2011 [cited 2021 03 June]; https://www.who.int/cardiovascular_diseases/publications/atlas_cvd/en/.

[2] Roth, G.A., G.A. Mensah, and V. Fuster, *The global burden of cardiovascular diseases and risks*: *A compass for global action*. 2020, American College of Cardiology Foundation Washington DC. p. 2980–2981. 10.1016/j.jacc.2020.11.021 33309174

[3] World Health Organization. *Hearts*: *Technical package for cardiovascular disease management in primary health care*. 2016 [cited 2020 3rd October 2020]; https://www.who.int/cardiovascular_diseases/hearts/Hearts package.pdf?ua=1.

[4] World Bank. *New World Bank country classifications by income level*: *2022–2023*. 2022 [cited 2023 15/05]; https://datahelpdesk.worldbank.org/knowledgebase/articles/906519-world-bank-country-and-lending-groups.

[5] Seidman, G., *Achieving universal primary health care through health systems strengthening*: *Lesotho’s national primary health care reform*, in *School of Public Health*. 2017, Harvard University Boston.

[6] World Health Organisation. *Non-communicable diseases country profiles* 2018 [cited 2022 11th July]; https://www.who.int/publications/i/item/9789241514620.

[7] World Bank. *Lesotho—cause of death*, *by non-communicable diseases (% of total)*. 2021 [cited 2023 18th July]; https://tradingeconomics.com/lesotho/cause-of-death-by-non-communicable-diseases-percent-of-total-wb-data.html.

[8] Ministry of Health (Lesotho). *National multi-sectoral integrated strategic plan for the prevention and control of non-communicable diseases*: *2014–2020*. 2014 [cited 2022 10th September]; https://www.iccp-portal.org/system/files/plans/LSO_B3_Endorsed%20NCD%20Stratergic%20Plan%20-%20Copy.pdf.

[9] World Health Organization. *Package on essential noncommunicable (PEN) disease interventions for primary health care*. 2020 [cited 2021 3rd June]; https://www.who.int/publications/i/item/who-package-of-essential-noncommunicable-(pen)-disease-interventions-for-primary-health-care.

[10] International Pharmaceutical Federation. *Community pharmacy section vision 2020–2025*. 2020 [cited 2022 8th November]; https://www.fip.org/files/CPS_vision_FINAL.pdf.

[11] Manouchehri M., Fernández-Alfonso M.S., and Gil-Ortega M., Impact of intervention of community pharmacists on cardiovascular outcomes in Spain: A systematic review. Journal of Pharmacy & Pharmacognosy Research, 2022. 10(5): p. 952–976.

[12] Cheema E., Sutcliffe P., and Singer D.R., The impact of interventions by pharmacists in community pharmacies on control of hypertension: A systematic review and meta-analysis of randomized controlled trials. Br. J. Clin. Pharmacol., 2014. 78(6): p. 1238–1247. doi: 10.1111/bcp.12452 24966032 PMC4256613

[13] Chiazor E.I., et al., A systematic review of community pharmacists’ interventions in reducing major risk factors for cardiovascular disease. Value in Health Regional Issues, 2015. 7: p. 9–21. doi: 10.1016/j.vhri.2015.03.002 29698158

[14] Motlohi N.F., et al., A systematic review of the role of community pharmacists in the prevention and control of cardiovascular diseases: the perceptions of patients. Systematic Reviews, 2023. 12(1): p. 160. doi: 10.1186/s13643-023-02338-7 37705090 PMC10500864

[15] Ministry of Health and Social Welfare (Lesotho), *Lesotho pharmaceutical country profile*., M.o.H.a.S. Welfare, Editor. 2011: Maseru.

[16] Park Y.S., Konge L., and Artino A.R., The positivism paradigm of research. Academic Medicine, 2020. 95(5): p. 690–694. doi: 10.1097/ACM.0000000000003093 31789841

[17] Motlohi N.F., et al., The role of Lesotho community pharmacists in preventing and controlling cardiovascular diseases: The perceived facilitators and barriers. PLOS ONE, 2024. 19(4). doi: 10.1371/journal.pone.0301525 38574015 PMC10994300

[18] Central Intelligence Agency. *The world factbook*. 2020 [cited 2020 14th April 2020]; https://www.cia.gov/library/publications/the-world-factbook/geos/print_lt.html.

[19] Lesotho Bureau of Statistics. *Population and housing census 2016*. 2020 [cited 2020 18th June]; https://catalog.ihsn.org/catalog/8293/related-materials.

[20] Ihekoronye M.R. and Osemene K.P., Evaluation of the participation of community pharmacists in primary healthcare services in Nigeria: a mixed-method survey. International Journal of Health Policy and Management, 2022. 11(6): p. 829–839. doi: 10.34172/ijhpm.2020.224 33300774 PMC9309912

[21] Sendekie A.K. and Netere A.K., Multicenter cross-sectional study on perceptions and roles of community pharmacists in the prevention and management of cardiovascular disorders in Northwest Ethiopia. Integrated Pharmacy Research and Practice, 2022: p. 21–31. doi: 10.2147/IPRP.S348260 35083130 PMC8784253

[22] Tlali, R., *Evaluation of the profile and standard of community pharmacy practice in Lesotho*. 2021, North-West University (South-Africa).

[23] Israel, G.D., *Determining sample size*. 1992.

[24] Vanderstoep S.W. and Johnson D.D., *Research Methods for Everyday Life*: *Blending Qualitative and Quantitative Approaches*. Vol. 32. 2008: John Wiley & Sons.

[25] Ajzen I., The Theory of Planned Behavior. Organizational Behavior and Human Decision Processes, 1991. 50(2): p. 179–211.

[26] Wagner J., et al., Development of a questionnaire to measure heart disease risk knowledge in people with diabetes: The Heart Disease Fact Questionnaire. Patient Education and Counseling, 2005. 58(1): p. 82–87. doi: 10.1016/j.pec.2004.07.004 15950840

[27] Cronbach L.J., Coefficient alpha and the internal structure of tests. psychometrika, 1951. 16(3): p. 297–334.

[28] Campbell M.J., Machin D., and Walters S.J., *Medical statistics*: *a textbook for the health sciences*. 2010: John Wiley & Sons.

[29] International Business Machines Corporation. *IBM SPSS software*. 2022 [cited 2021 12th November]; https://www.ibm.com/spss.

[30] Sulaiteen F.M., et al. Awareness of Cardiovascular Disease Risk Factors by Community Pharmacists in Saudi Arabia. in *Healthcare*. 2023. MDPI. doi: 10.3390/healthcare11020151 36673520 PMC9859281

[31] Nsengimana A., et al., Attitudes, perceptions, and barriers of community pharmacists in Rwanda towards health promotion: a cross sectional study. Archives of Public Health, 2022. 80(1): p. 157. doi: 10.1186/s13690-022-00912-4 35733223 PMC9217721

[32] Microsoft Corporation. *Microsoft Excel*. 2013; https://www.microsoft.com/en-us/?ql=4.

[33] Bennett D.A., How can I deal with missing data in my study? Australian and New Zealand journal of public health, 2001. 25(5): p. 464–469. 11688629

[34] Dong Y. and Peng C.-Y.J., Principled missing data methods for researchers. SpringerPlus, 2013. 2: p. 1–17. 23853744 10.1186/2193-1801-2-222 PMC3701793

[35] Tabachnick B.G., Fidell L.S., and Ullman J.B., *Using multivariate statistics*. Vol. 6. 2013: pearson Boston, MA.

[36] Motlohi N.F., Wiafe B., Mensah K.B., Padayachee N., Petrus R., & Bangalee V., A systematic review of the role of community pharmacists in the prevention and control of cardiovascular diseases: the perceptions of patients. Systematic Reviews, 2023. 12(160). 10.1186/s13643-023-02338-7 PMC10500864 37705090

[37] Henneh A.H. and Teg-Nefaah Tabong P., Community pharmacists perception and role in the prevention and management of cardiovascular disease conditions: Evidence from Ghana. The International Journal of Health Planning and Management, 2022. 37(5): p. 2794–2808. doi: 10.1002/hpm.3504 35607292

[38] Ministry of Health (Lesotho), *Standard Treatment Guidelines for Lesotho*, M.o. Health, Editor. 2017: Maseru.

[39] Asmelashe Gelayee D., Binega Mekonnen G., and Asrade Atnafe S., Practice and barriers towards provision of health promotion services among community pharmacists in Gondar, Northwest Ethiopia. BioMed Research International, 2017. 2017. doi: 10.1155/2017/7873951 28831398 PMC5555023

[40] Sam A.T. and Parasuraman S., The nine-star pharmacist: An overview. Journal of Young pharmacists, 2015. 7(4): p. 281.

[41] Amadi C.E., et al., Knowledge of cardiovascular disease risk factors and practice of primary prevention of cardiovascular disease by community pharmacists in Nigeria: A cross-sectional study. Int. J. Clin. Pharm., 2018. 40(6): p. 1587–1595. doi: 10.1007/s11096-018-0744-3 30474770 PMC6280866

[42] Birarra M.K., et al., Knowledge of cardiovascular disease risk factors, practice, and barriers of community pharmacists on cardiovascular disease prevention in North West Ethiopia. Metabolism Open, 2022. 16: p. 100219. doi: 10.1016/j.metop.2022.100219 36466186 PMC9712979

[43] Santschi V., et al., Team-based care for improving hypertension management: A pragmatic randomized controlled trial. Frontiers in Cardiovascular Medicine, 2021. 8. doi: 10.3389/fcvm.2021.760662 34760950 PMC8572997

[44] Alavudeen S.S., et al. Cardiovascular disease-related health promotion and prevention services by pharmacists in Saudi Arabia: How well are they prepared? in Healthcare. 2023. MDPI. doi: 10.3390/healthcare11111614 37297754 PMC10252446

[45] Jahangard-Rafsanjani Z., et al., Pharmacists’ attitudes and perceived barriers about community pharmacy-based cardiovascular risk screening services. Journal of Pharmaceutical Care, 2014: p. 142–148.

[46] The Parliament of Lesotho, *Lesotho medicines and medical devices control act*. 2023, The Authority of His Majesty the King: Maseru.

[47] El Hajj M.S., Abu Yousef S.E., and Basri M.A., Diabetes care in Qatar: a survey of pharmacists’ activities, attitudes and knowledge. International Journal of Clinical Pharmacy, 2018. 40: p. 84–93. doi: 10.1007/s11096-017-0562-z 29147964

[48] Al-Ashwal F.Y., et al., Knowledge, attitude, perceived barriers, and practices among pharmacists regarding risk assessment of cardiovascular disease: A cross-sectional study in Yemen. Current medical research and opinion, 2022. 38(3): p. 451–459. doi: 10.1080/03007995.2021.1994380 34657524

[49] Cannon-Breland, M.L., *An Exploratory Study of Pharmacist Self-Reported Antidepressant Medication Counseling Behaviors*. 2011, Auburn University: United States—Alabama. p. 210.

[50] Witry, M.J., *Community pharmacist medication monitoring attitudes and decision making*. 2013, The University of Iowa: United States—Iowa. p. 217.

[51] Grujicic S. and Nikolic A., Cross-section studies: advantages and disadvantages. Zdravstvena zaštita, 2021. 50: p. 44.

[52] Stoltzfus J.C., Logistic Regression: A Brief Primer. Academic Emergency Medicine, 2011. 18: p. 1099–1104. doi: 10.1111/j.1553-2712.2011.01185.x 21996075

