# Supplementary material for: A multicenter cross-sectional survey of the role of community pharmacists, attitudes, and perceptions in preventing and controlling cardiovascular diseases
Source: PLoS One. 2025 Feb 10;20(2):e0314487. doi: 10.1371/journal.pone.0314487 (PMC11809882; doi:10.1371/journal.pone.0314487)
Supplement: S2 Table — (PDF) [file pone.0314487.s002.pdf]

THE ROLE OF COMMUNITY PHARMACISTS IN THE PREVENTION AND CONTROL OF CARDIOVASCULAR DISEASES IN LESOTHO

* Indicates required question

SECTION A: PARTICIPANT SOCIO-DEMOGRAPHIC INFORMATION

1.0 Pharmacy name where you are working as a community pharmacist

Your answer

1.1 Gender

- Female
- Male
- Prefer not to say

1.2 How old are you

- 18-29 years
- 30-44 years
- 45-59 years
- 60-69
- 70+ years

Pre-fill responses, then click "Get link"

1.3 Years of experience as a community pharmacist

- Less than 6 months
- 6 months to 1 year
- 1 year to 4 years
- 5 years to 9 years
- 10+ years

1.4 Educational Level

- Bachelor of Pharmacy/honours
- Masters in Pharmacy
- PhD in Pharmacy/Equivalent
- Other:

1.5 Employment status (tick all that applies)

- Pharmacy-Owner and full time employee
- Pharmacy-owner and part time employee
- Full time employee
- Part time employee

1.6 Working hours per week

Your answer

Pre-fill responses, then click "Get link"

2.0 PHARMACY SETTING

2.10 Location of the pharmacy

- ○ CBD/Urban
- ○ Suburban (Within local public transport distance from the city)
- ○ Rural (outside of local public transport distance from the city)

2.20 Type of Pharmacy

- ○ Independent
- ○ Chain store
- ○ Other:

2.30 Pharmacy operating hours from Monday to Friday

Your answer

2.40 Pharmacy working hours on weekends

Your answer

2.50 Categories of other pharmacy employees & number

Your answer

Pre-fill responses, then click "Get link"

2.60 Average number of patients with CVDs risk factors and established CVDs seen per day

Your answer

2.70 Did you receive CVDs education during your undergraduate pharmacy program?

- ○ Yes
- ○ No

2.80 Have you ever attended CVDs management course as a continuous professional development?

- ○ Yes
- ○ No

2.90

If you would like to receive a report of the study findings, please provide your email address

Your answer

SECTION B: Role of a community Pharmacist in prevention and control of cardiovascular diseases (CVDs).

Pre-fill responses, then click "Get link"

3.0 Please Indicate how often you provide each of the following services at your pharmacy to CVDs patients. *

	Never	Rarely	Sometimes	Often	Always
Dispensing prescription only medicine	○	○	○	○	○
Dispensing over-the-counter medicine (e.g. supplements)	○	○	○	○	○
Screening for Hypertension	○	○	○	○	○
Screening for Diabetes	○	○	○	○	○
Screening for hyperlipidemia (cholesterol)	○	○	○	○	○
Screening for undiagnosed CVDs (heart attacks and strokes)	○	○	○	○	○
Alcohol abuse management	○	○	○	○	○
Smoking cessation advice	○	○	○	○	○
Weight management advice	○	○	○	○	○
Keeping patient medical record database	○	○	○	○	○
Patient self-care advice (Personal testing Record)	○	○	○	○	○
[illegible] CVDs education					

Pre-fill responses, then click "Get link"

Medication use review/Drug utilisation review	○	○	○	○	○
Referral of the patient	○	○	○	○	○
Follow up on patients	○	○	○	○	○
Identify drug-related problems	○	○	○	○	○
Make appointments with patients	○	○	○	○	○
Disseminate disease information to patients (e.g. pamphlets)	○	○	○	○	○
Inter-professional communication regarding the patient health	○	○	○	○	○

SECTION C: CVDs risk factors

Pre-fill responses, then click "Get link"

4.0 Please indicate whether you agree (True) or disagree (False) with the following statements. Tick 'I don't know' if you do not know. *

	True	False	I don't know
A person is always aware when they have CVDs	○	○	○
Hyperlipidaemia (high cholesterol) increases chances of CVDs	○	○	○
The risk of CVDs increases with age	○	○	○
Controlled blood pressure will reduce a person' risk of CVDs progression	○	○	○
A diabetic patient can reduce his/her risk of CVDs if his/her cholesterol level is controlled	○	○	○
Diabetic patients often have low HDL cholesterol	○	○	○
Regular physical exercise will lower risk of CVDs	○	○	○
Overweight and obesity are risk factors of CVDs	○	○	○
A fatty meal has no effects on blood cholesterol levels	○	○	○
A person who smokes is at risk of CVDs	○	○	○
[illegible] factor for CVDs			

Pre-fill responses, then click "Get link"

High blood sugar makes the heart work harder

Hypertension is a risk factor of CVDs

Diabetic patients hardly have high cholesterol

Family history of heart disease increases a risk of heart disease

Gender is a risk factor for CVDs progression in patients with diabetes

A person whose HDL is high is at risk for CVDs

Prolonged increase in blood sugar levels can trigger increase in cholesterol and increased chances of CVDs

A diabetic patient can reduce his/her risk of getting CVDs if his/her weight is kept under control

A person whose LDL is high is at risk of CVDs

A diabetic patient can reduce his/her chances of getting CVDs if his/her sugar blood levels is kept

Pre-fill responses, then click "Get link"

A diabetic patient [illegible]

can reduce his/her chances of getting CVDs if his/her blood pressure levels is kept under control ○ ○ ○

Smoking cessation lowers risk of developing CVDs ○ ○ ○

Physical exercise will lower a person's chances of CVDs if it is taken only at a gym or under coached by an instructor ○ ○ ○

Walking and gardening are not considered exercise that will lower risk of developing CVDs ○ ○ ○

coached by an instructor

Walking and gardening are not considered exercise that will lower risk of developing CVDs ○ ○ ○

SECTION D: Enhancing community pharmacist role in the prevention and control of CVDs

Pre-fill responses, then click "Get link"

5.0 Please indicate to what extend you agree or disagree with the following statements to indicate how community pharmacist's role in the prevention and control of CVDs could be enhanced. Tick the appropriate boxes. *

	Strongly disagree	Disagree	Neither agree nor disagree	Agree	Strongly agree
Enhancing the public health content of the pharmacy training curricula	○	○	○	○	○
Remunerating pharmacists for providing health promotion services	○	○	○	○	○
Availability of and familiarity with guidelines by community pharmacists	○	○	○	○	○
Availability of regulations and policies that incorporates community pharmacist's participation in these activities	○	○	○	○	○
Completion of postgraduate internship program should become a pre-requisite for pharmacist registration.	○	○	○	○	○
Awareness of the role of community pharmacists to [illegible]	○	○	○	○	○

Pre-fill responses, then click "Get link"

Close monitoring

and enforcement of compliance by the regulatory authority/Ministry of Health	○	○	○	○	○
Continuous Professional Development programs for community pharmacists	○	○	○	○	○

pharmacists

Pre-fill responses, then click "Get link"

6.0 What additional services do you think community pharmacists could provide in the future for prevention and control of CVDs? *

	Strongly disagree	Disagree	Neither agree nor disagree	Agree	Strongly agree
Utilize various platforms (e.g. pamphlets, social media, radios & TVs) to educate the public about CVDs	○	○	○	○	○
Diagnose and prescribe at a fee	○	○	○	○	○
Create patient groups to encourage lifestyle modification	○	○	○	○	○
Community outreach services	○	○	○	○	○
Increase screening capacity services	○	○	○	○	○
Monitoring and review of treatment	○	○	○	○	○
What we are currently doing is enough	○	○	○	○	○
Use of advanced testing devices	○	○	○	○	○

Pre-fill responses, then click "Get link"

7.0 Please indicate to what extend you agree or disagree with the following statements about factors that may affect your provision of health promotion services to hypertension (HTN) patients by ticking the appropriate box *

	Strongly disagree	Disagree	Neither agree nor disagree	Agree	Strongly agree
HTN patients' inability to obey medication adherence instructions leads to unfavourable outcomes	○	○	○	○	○
Patients' lack of awareness of community pharmacists' services is a barrier to the provision of services to patients	○	○	○	○	○
Lack of time by the patient is a barrier to the provision of services to patients	○	○	○	○	○
Lack of interest by HTN patients is a barrier to the provision of services to HTN patients	○	○	○	○	○
Lack of communication between community pharmacists and other healthcare providers is a [illegible] to HTN patients					

Pre-fill responses, then click "Get link"

Lack of integration between community pharmacy-based services and other healthcare providers is a barrier to the provision of health promotion services to HTN patients	○	○	○	○	○
Lack of personnel and tools/equipment is a barrier to the provision of services to patients	○	○	○	○	○
The socio-economic status of patients discourages HTN patients from satisfactorily adhering to their medication	○	○	○	○	○
It is difficult to get people to avoid CVD risk behaviours/to engage in healthy lifestyles	○	○	○	○	○
Fragmented supply chain regulations hinder my full potential to provide healthcare services to HTN patients	○	○	○	○	○

Pre-fill responses, then click "Get link"

discourages me

[illegible]

from providing health promotion services to HTN patients

I feel uncomfortable asking patients about their CVD risk factors, e.g. smoking

I believe I do not have adequate knowledge and skills in CVD management

I am not interested in providing health promotion services to HTN patients

Lack of electricity at my pharmacy location forces me to knock off earlier than would be preferred]

Health promotion services to HTN patients are not the role of community pharmacists

My relationship with other community pharmacists is good

I have enough [illegible]

Pre-fill responses, then click "Get link"

counselling to

every single HTN patient	○	○	○	○	○
My relationship with physicians involved in cardiovascular care motivates me to provide medication counselling and weight loss advice to HTN patients	○	○	○	○	○
HTN patients are always willing to receive my medication and weight advise	○	○	○	○	○
There is enough support from the regulatory authority (Ministry of Health) that encourages my provision of medication adherence counselling to HTN patients	○	○	○	○	○
My pharmacy has a sufficient number of staff to provide medication adherence counselling to HTN patients	○	○	○	○	○
A private area is available at my pharmacy to provide counselling	○	○	○	○	○

Pre-fill responses, then click "Get link"

counselling to HTN patients

A private area is available at my pharmacy to provide counselling ○ ○ ○ ○ ○

SECTION F: Community pharmacists attitude and perceptions towards their role in prevention and control of CVDs

Pre-fill responses, then click "Get link"

8.0 Please indicate to what extend you agree or disagree with the following statements by ticking the appropriate box *

	Strongly diasgree	Disagree	Neither agree nor disagree	Agree	Strongly agree
If I provide health promotion services (such as lifestyle modification) to HTN patients, I feel that I am doing something positive for the patients	○	○	○	○	○
Patient self-monitoring of CVD risk factors forms part of community pharmacist services	○	○	○	○	○
Involvement of a community pharmacist in the primary healthcare framework in the prevention and control of CVDs can result in satisfactory disease outcome	○	○	○	○	○
Integrating CVD health promotion into community pharmacy daily practice is [illegible] CVD prevention	○	○	○	○	○

Pre-fill responses, then click "Get link"

and control should form part of community pharmacy practice standards in Lesotho	○	○	○	○	○
CVD health promotion is part of community pharmacy practice standard	○	○	○	○	○
Physicians believe that I should advise HTN patients on lifestyle modification	○	○	○	○	○
It is my employment obligation that I provide HTN patients with lifestyle modification	○	○	○	○	○
HTN Patients appreciate the pharmacist's effort to counsel them	○	○	○	○	○
I am confident that I can counsel HTN patients on lifestyle modification	○	○	○	○	○
Providing lifestyle modification counselling to [illegible]					

Pre-fill responses, then click "Get link"

I am able to [illegible]

I am able to initiate lifestyle counselling on my own decision ◯ ◯ ◯ ◯ ◯

easy

I am able to initiate lifestyle counselling on my own decision ◯ ◯ ◯ ◯ ◯

SECTION G: Cost associated with community pharmacy roles to CVDs patients:

On average, how much do the following services cost?

9.0 Smoking cessation advice

Your answer

10.0 Weight management counselling

Your answer

11.0 Screening for Hypertension

Your answer

Pre-fill responses, then click "Get link"

12.0 Screening for hyperlipidemia (cholesterol)

Your answer

13.0 Screening for undiagnosed CVDs (heart attacks and strokes)

Your answer

14.0 Screening for Diabetes

Your answer

15.0 Alcohol abuse management

Your answer

16.0 Smoking cessation advice

Your answer

17.0 Patient self-care advice (Personal testing Record)

Your answer

Pre-fill responses, then click "Get link"

18.0 CVDs education

Your answer

19.0 Medication use review/Drug utilization review

Your answer

20.0 Referral of the patient

Your answer

21.0 Follow up on patients

Your answer

22.0 Identify drug-related problems

Your answer

Pre-fill responses, then click "Get link"

23.0 Make appointments with patients

Your answer

24.0 Disseminate disease information to patients (e.g. pamphlets)

Your answer

SECTION H: Availability of tools

25.0 Indicate the availability of the following items in your facility clinic/consulting room by marking Available to show presence, Not available to show absence. *

	Available	Not available
Blood pressure measurement device	○	○
Measuring tape	○	○
Height measuring device	○	○
Body Weight scale/balance	○	○
Blood sugar measuring device (Glucometer)	○	○
Blood cholesterol measuring device	○	○
Standard treatment guidelines for Lesotho	○	○

Pre-fill responses, then click "Get link"

Feedback/recommendations/Suggestions

Thank you for your time

Get link

Never submit passwords through Google Forms.

This content is neither created nor endorsed by Google. Report Abuse - Terms of Service - Privacy Policy

Google Forms

Pre-fill responses, then click "Get link"

Pre-fill responses, then click "Get link"
